# Discrepancies in gut microbial communities and serum metabolites of Hu sheep with different backfat thickness

**DOI:** 10.3389/fmicb.2025.1667088

**Published:** 2025-10-16

**Authors:** Bo Li, Wenwen Xu, Wenjia Wang, Mengyuan Mao, Xiaoyu Huang, Enping Zhang

**Affiliations:** College of Animal Science and Technology, Northwest A and F University, Xianyang, China

**Keywords:** backfat thickness, metagenomics, serum metabolomics, gut microbiota, sheep

## Abstract

Although market demand for lean meat continues to rise, the regulatory mechanisms governing backfat thickness (BFT) metabolism remain poorly understood. This study employed a multi-omics approach to investigate BFT-associated differences in Hu sheep with distinct fat deposition phenotypes. From 160 genetically similar Hu sheep, we selected 12 individuals with non-significant weight differences (*P* > 0.05) but extreme divergence in BFT [6 high-BFT (HBF) and 6 low-BFT (LBF) individuals]. Using integrated metagenomics and metabolomics, we systematically compared ileal microbial community structure and serum metabolic profiles between the two groups. HBF sheep showed significantly increased adiposity and altered ileal microbiota composition, characterized by elevated abundances of *Carnobacterium, Parabacteroides distasonis, Lactiplantibacillus*, and *Bifidobacterium*. Serum metabolomics identified key differential glycerophospholipids-1-(9Z-octadecenoyl)-2-(11Z-eicosenoyl)-glycero-3-phosphate, PE-NMe(15:0/20:3(5Z,8Z,11Z)), PE-NMe_2_(18:1(9Z)/20:0), and PE-NMe_2_(18:1(9Z)/22:1(13Z))-all enriched in glycerophospholipid metabolism pathways. Integrated correlation analysis revealed strong associations between *P. distasonis* abundance and these phospholipids. These results demonstrate BFT-related adaptive remodeling of the serum metabolome and gut microbiota, identifying *P. distasonis* as a potential modulator of the host-microbe metabolic axis in ovine adiposity regulation.

## 1 Introduction

Fat is an essential nutrient in humans and animals, regulating life activities through the absorption of glucose and fatty acids and the secretion of diverse bioactive molecules, including hormones, metabolites, and genetic material (Rosen and Spiegelman, 2014). However, dysregulation of fat secretion can lead to metabolic disorders such as lipid overload, inflammation, and organelle stress, posing risks to health (Julibert et al., 2019; An et al., 2023). Over the long process of domestication, sheep have evolved to deposit substantial fat reserves in subcutaneous tissue and the tail, serving as adaptations to withstand harsh environments and store energy (Cheng et al., 2016). In modern intensive sheep farming, excessive subcutaneous fat deposition diminishes lean meat yield and impairs animal health, increasing vulnerability to metabolic disorders, inflammation, and reduced disease resistance (Liu et al., 2024). Concurrently, rising consumer health awareness has intensified scrutiny of the link between dietary fat intake and cardiovascular disease risk (Schlesinger et al., 2019; Sacks et al., 2017). Consequently, negative perceptions of high-fat meat products are driving increased market demand for leaner alternatives, presenting new challenges for sheep producers (Diekman and Malcolm, 2009). These converging factors make the regulation of ovine fat deposition a paramount goal for breeders globally.

In livestock production, Backfat Thickness (BFT) serves as a key indicator of ovine fat deposition. Excessive BFT (> 20 mm) diminishes carcass economic value and may impose a greater metabolic burden on the animal, thereby reducing production efficiency (Zhou et al., 2018). Multiple factors contribute to elevated BFT in sheep, encompassing genetics, diet, and management practices (Du et al., 2022; Zhou et al., 2024; Cerdeño et al., 2006). Serum metabolomics offers a novel perspective for investigating fat deposition, enabling further elucidation of fat metabolism molecular mechanisms through metabolite analysis (Bovo et al., 2016). For instance, Yu et al. (2023) utilized serum metabolomics in Qinchuan cattle divergent for BFT, identifying key sphingolipid metabolites implicated in the systemic regulation of backfat thickness. Currently, research on the impact of BFT on ovine meat quality and adipogenesis remains limited, warranting further exploration into the molecular mechanisms underlying ovine lipid deposition.

The intestinal microbiota significantly modulates lipid metabolism, through diverse mechanisms, it influences lipid absorption, synthesis, and breakdown, thereby regulating host adipogenesis (Moszak et al., 2020; Hou et al., 2024). Research indicates that gut microbes enhance lipid absorption and storage by suppressing the expression of the long non-coding RNA Snhg9 (Wang et al., 2023). Moreover, alterations in microbial community composition and function can direct the efficiency and trajectory of lipid metabolism. Supporting this, Kang et al. (2024) investigated correlations between gut microbiota and phenotypic traits in Sunit sheep under varying feeding regimens. Their findings suggest that shifts in lipid metabolism may be partially linked to differences in bacterial populations.

This study is premised on the complex crosstalk between host metabolism and intestinal microbiota composition (Li et al., 2023; Yang et al., 2024). We hypothesize that the compositional profile and metabolic output of the ileal microbiota in Hu sheep modulate host adipogenesis via serum metabolic mediators, consequently influencing backfat thickness (BFT). To test this hypothesis, we employed integrated metagenomic and metabolomic analyses to characterize differences in ileal microbial community structure and associated metabolites in Hu sheep cohorts with shared genetic backgrounds and body weights, but divergent BFT phenotypes. This research aims to elucidate the mechanisms through which microbial community composition and metabolic activity govern host lipid metabolism.

## 2 Materials and methods

### 2.1 Experimental animals and experimental design

**Figure 1:**
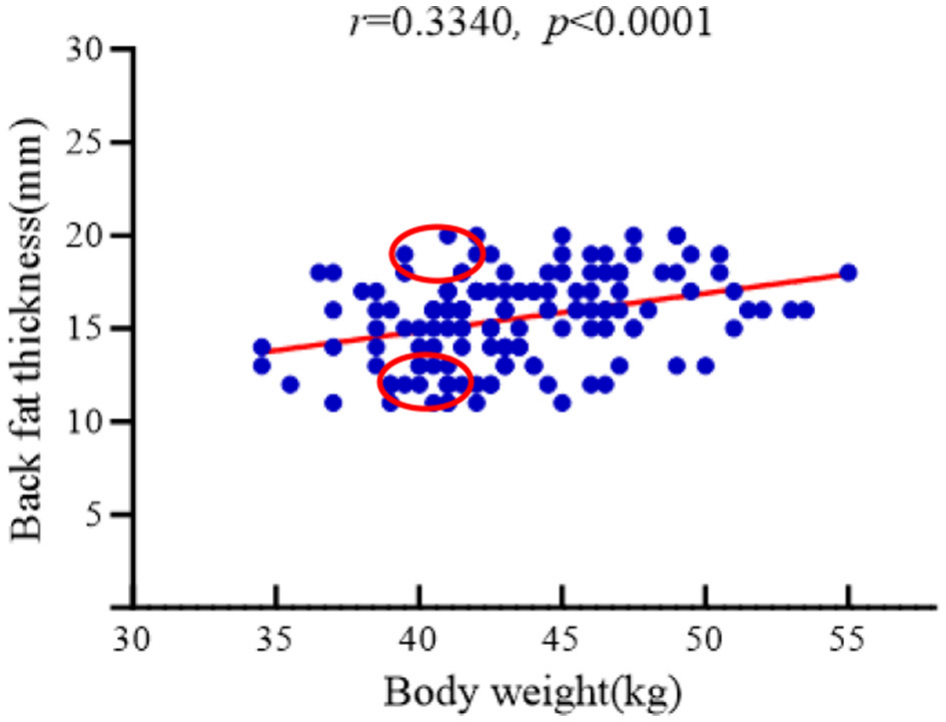
Correlation analysis between body weight and backfat thickness of Hu sheep.

A total of 160 unsold Hu sheep ewes (8-month-old) from Xinzhongsheng Sheep Farm (Yulin City, Shaanxi Province) were subjected to body the weight and backfat thickness measurements. Backfat thickness was measured using A-mode ultrasonography (Renco Lean-Meater^®^, Minneapolis, MN, USA). To examine the relationship between these parameters, body weight data were visualized through scatter plots and analyzed via linear regression. Figure 1 demonstrates a statistically significant positive correlation between backfat thickness and body weight (r = 0.3340, *P* < 0.001), with both variables adhering to normal distribution patterns. From the cohort of 160 sheep, two experimental groups were established under same body weight (41 ± 0.5 kg) conditions: High Backfat (HBF) group (Backfat thickness = 19 ± 0.5 mm; n = 6) and Low Backfat (LBF) group (Backfat thickness = 11 ± 0.5 mm; n = 6). The dietary information for all participating Hu sheep is presented in Table 1.

**Table 1:** Composition and nutrient levels of basal diets.

**Items**	**Concentrate supplement**	**Alfalfa hay**
**Ingredients %**		
Corn	61.54	
Soybean meal	12.32	
Wheat bran	6.15	
DDGS ^a^	15.38	
Premix ^b^	2.69	
CaHPO_4_	0.92	
NaHCO_3_	0.54	
NaCl	0.31	
Desorbent	0.15	
Total	100.00	
**Nutrition levels** ^c^ **%**		
Dry matter	90.46	90.94
Crude protein	19.5	7.93
Ether extract	2.8	0.76
Neutral detergent fiber	17.84	53.62
Acid detergent fiber	7.04	31.49
Phosphorus	1.14	0.27
calcium	1.91	2.01
Gross energy, MJ/Kg	16.84	16.70

^a^ DDGS stands for Distillers dried grains with soluble.

^b^ The premix provided the following per kg of diet: vitamin A, 3,50,000 IU; biotin, 50 mg; manganese, 1,300 mg; cobalt, 15 mg; vitamin D3: 70,000 IU; niacinamide, 550 mg; zinc, 1,500 mg; calcium, 10%; vitamin E, 1,100 IU; copper, 180 mg; iodine, 20 mg; sodium chloride, 16%; vitamin B6, 1,000 mg; iron, 500 mg; selenium: 8 mg.

^c^ All nutritional components in the experimental diets were measured according to Chinese National Standards (GB/T). Specific analytical methods included: dry matter (GB/T 6435–2014), crude protein (GB/T 6432–2018), neutral/acid detergent fiber (GB/T 6434–2006), crude fat (GB/T 6433–2006), crude ash (GB/T 6438–2022), calcium (GB/T 6436–2018), and phosphorus (GB/T 6437–2018). Gross energy content was determined using a C6000 bomb calorimeter (Germany).

### 2.2 Sample collection

On the morning following the completion of the trial period, ileum contents were collected from the 12 Hu sheep in both the HBF and LBF groups. Immediately transfer approximately 10 g of ileal digestate sample into liquid nitrogen for rapid freezing, then store the frozen specimen at −80°C until required for subsequent analytical procedures. Then, in order to balance experimental costs and necessary repetition times, six ileal contents samples were selected from each group for further metabolomic analysis.

### 2.3 Serum sampling and analysis

Blood samples were collected with non-anticoagulation vacuum blood vessels before morning feeding after a 12-hr fast the day before slaughter. Serum was harvested following centrifugation at 3,000 × g for 10 min at 4°C and subsequently frozen at −80°C until analysis. The centrifuge model used in this process is TDL-80–2B, manufactured in Shanghai, China.

The concentrations of serum albumin (ALB), alkaline phosphatase (ALP), alanine aminotransferase (ALT), aspartate aminotransferase (AST), total cholesterol (CHO), high density lipoprotein (HDL), low density lipoprotein (LDL), triglycerides (TG), total protein (TP), γ-glutamylaminotransferase (γ-GT) and superoxide dismutase (SOD) were determined using corresponding commercial kits (Zhongsheng Beikong Bio-technology and Science Inc., Beijing, China) and an automatic biochemical analyzer (BS-420, Shenzhen Mindray Bio-medical Electronics Co., Shenzhen, China).

### 2.4 Metabolic analysis based on liquid chromatography-mass spectrometry (LC-MS)

The sample stored at −80°C was thawed on ice and vortexed for 10 s. A 50 μL aliquot of the sample and 300 μL of extraction solution (methanol: acetonitrile = 1:4, V/V) containing internal standards were added into a 2 mL microcentrifuge tube. The mixture was vortexed for 3 min and then centrifuged at 12,000 rpm for 10 min at 4°C (The centrifuge model used in this process is TGL-16B, manufactured in Shanghai, China.). A 200 μL aliquot of the supernatant was collected and placed at −20°C for 30 min, followed by centrifugation at 12,000 rpm for 3 min at 4°C. A 180 μL aliquot of the supernatant was transferred for LC-MS analysis. Each sample was analyzed using two LC/MS methods. One aliquot was analyzed under positive ion conditions and eluted from a T3 column (Waters ACQUITY Premier HSS T3 Column, 1.8 μm, 2.1 mm × 100 mm) using 0.1% formic acid in water as solvent A and 0.1% formic acid in acetonitrile as solvent B with the following gradient: 5% to 20% in 2 min, increased to 60% in the next 3 min, increased to 99% in 1 min, held for 1.5 min, then returned to 5% mobile phase B within 0.1 min, and held for 2.4 min. The analytical conditions were as follows: column temperature, 40°C; flow rate, 0.4 mL/min; injection volume, 4 μL. The second aliquot was analyzed under negative ion conditions using the same elution gradient as the positive mode.

### 2.5 Microbial DNA isolation, metagenomic sequencing, and functional annotation

According to the manufacturer's standard protocol, microbial DNA from the ileal contents was isolated using the E.Z.N.A.^®^ Fecal DNA Kit (Omega Bio-tek Inc., Norcross, GA, USA). After genomic DNA extraction, the quality of the isolated DNA was evaluated by 1% agarose gel electrophoresis, and qualified DNA samples were stored at −80°C until subsequent sequencing.

A total of 1 μg of genomic DNA per sample was used as the starting material for library preparation. Sequencing libraries were generated using the NEBNext^®^ Ultra™ DNA Library Prep Kit for Illumina (NEB, USA) following the manufacturer's recommendations. Index codes were added to assign sequences to each sample. Briefly, we utilized the Diagenode Bioruptor^®^ UCD-300 TS non-contact focused ultrasound shearing device (Belgium) to fragment DNA samples into an average length of 350 base pairs (bp). DNA fragments were end-polished, A-tailed, and ligated with Illumina adapters. Further PCR amplification was performed to enrich the ligated DNA fragments. PCR products were purified using the AMPure XP system. Libraries were analyzed for size distribution using the Agilent 2,100 Bioanalyzer and quantified using real-time quantitative PCR (qPCR). The clustering of index-coded samples was performed on a cBot Cluster Generation System according to the manufacturer's instructions. After cluster generation, library preparations were sequenced on an Illumina Nova-Seq platform, and paired-end sequencing was performed to generate reads.

### 2.6 Microbiome and metabolomics

In the omics data analysis, n = 6 indicates six biological replicates were used. For between group differential analysis, Student's *t*-test was applied when homogeneity of variance was satisfied; otherwise, the Wilcoxon rank-sum test was used, with statistical significance defined at *P* < 0.05. For integrative analysis of microbial metagenomics and metabolomics, we systematically evaluated associations among three key elements through Spearman's rank correlation analysis: 1) the relative abundance of bacterial taxa within the microbial community, 2) previidentified microbial genera, and 3) standardized individual metabolic profiles.

### 2.7 Statistical analysis

Use one-way analysis of unpaired *t*-test for inter group statistical comparison. Data are expressed as means ± standard error of the mean (SEM). Statistical thresholds were designated as follows: *P* < 0.05 (significant) and 0.05 ≤ *P* < 0.10 (trend-level). All analyses and visualizations were conducted employing GraphPad Prism 8.0 (San Diego, USA).

## 3 Results

### 3.1 Serum biochemical indices

As summarized in Table 2, the HBF cohort demonstrated significantly higher HDL concentrations vs the LBF group (*P* < 0.05). Notably, the HBF group exhibited a trend toward elevated LDL and AST levels (0.05 < *P* < 0.10).

**Table 2:** Blood nutrients characteristics and antioxidant capacity of the Hu sheep.

**Items**	**HBF Group**	**LBF Group**	***P*-value**
ALB (g·L^−1^)	28.17 ± 3.57	26.60 ± 0.96	0.39
ALP (U·L^−1^)	224.83 ± 26.48	273.83 ± 19.95	0.17
ALT (U·L^−1^)	18.02 ± 1.92	13.53 ± 1.9	0.13
AST (U·L^−1^)	127.88 ± 11.89	100.55 ± 6.94	0.08
CHO (mmol·L^−1^)	1.71 ± 1.87	1.43 ± 0.07	0.18
HDL (mmol·L^−1^)	1.08 ± 0.08	0.85 ± 0.03	0.03
LDL (mmol·L^−1^)	0.77 ± 0.09	0.57 ± 0.04	0.09
TG (mmol·L^−1^)	0.18 ± 0.02	0.19 ± 0.02	0.82
TP (g·L^−1^)	69.27 ± 3.20	66.27 ± 1.37	0.41
γ-GT (U·L^−1^)	52.67 ± 3.05	54.50 ± 2.06	0.63
SOD (U·mL^−1^)	135.83 ± 13.20	136.50 ± 8.53	0.97

### 3.2 Serum metabolomic characteristics of groups with different BFT

**Figure 2:**
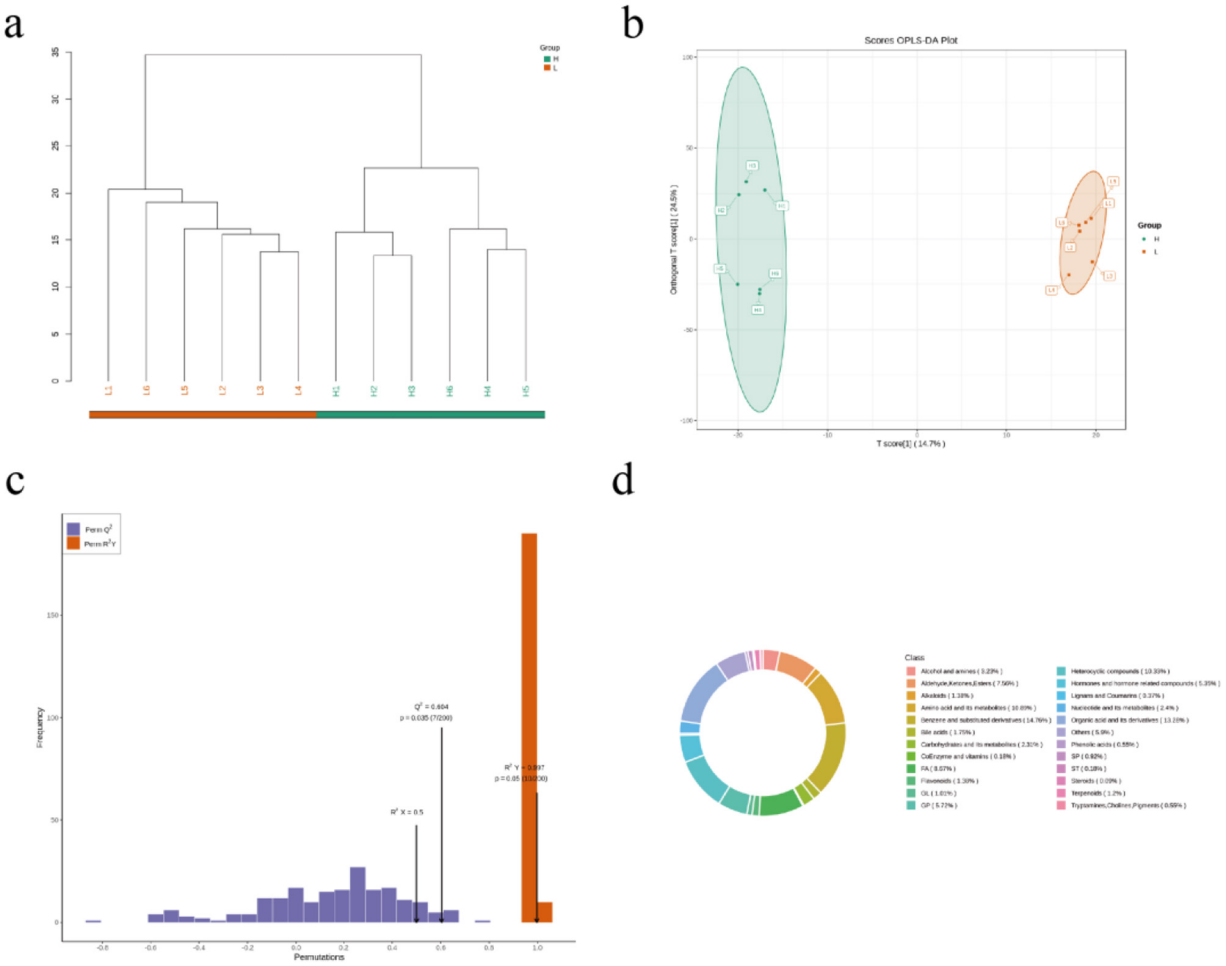
Identification and classification of serum metabolites from Hu sheep with high and low BFT. **(a)** cluster analysis plot of 12 samples **(b)** OPLS-DA score plot **(c)** OPLS-DA permutation test plot **(d)** metabolite classification spectra.

Untargeted serum metabolomic profiling of Hu sheep cohorts was performed using UHPLC-Q-Exactive MS to characterize metabolic disparities between divergent backfat thickness phenotypes (Figure 2a). Multivariate analyses revealed distinct intergroup clustering via PCA and PLS-DA, with model validity confirmed through permutation testing (Figure 2b). The OPLS-DA model demonstrated exceptional robustness (R^2^Y = 0.997, *P* = 0.05; Q^2^ = 0.604, *P* < 0.05), indicating high predictive fidelity (Figure 2c). The identified metabolites were compared with KEGG compound database and HMDB to obtain metabolite classification spectra (Figure 2d). We found that the majority of these metabolites are organic acids and their derivatives in each group, followed by organic heterocyclic compounds and amino acids and their derivatives.

**Figure 3:**
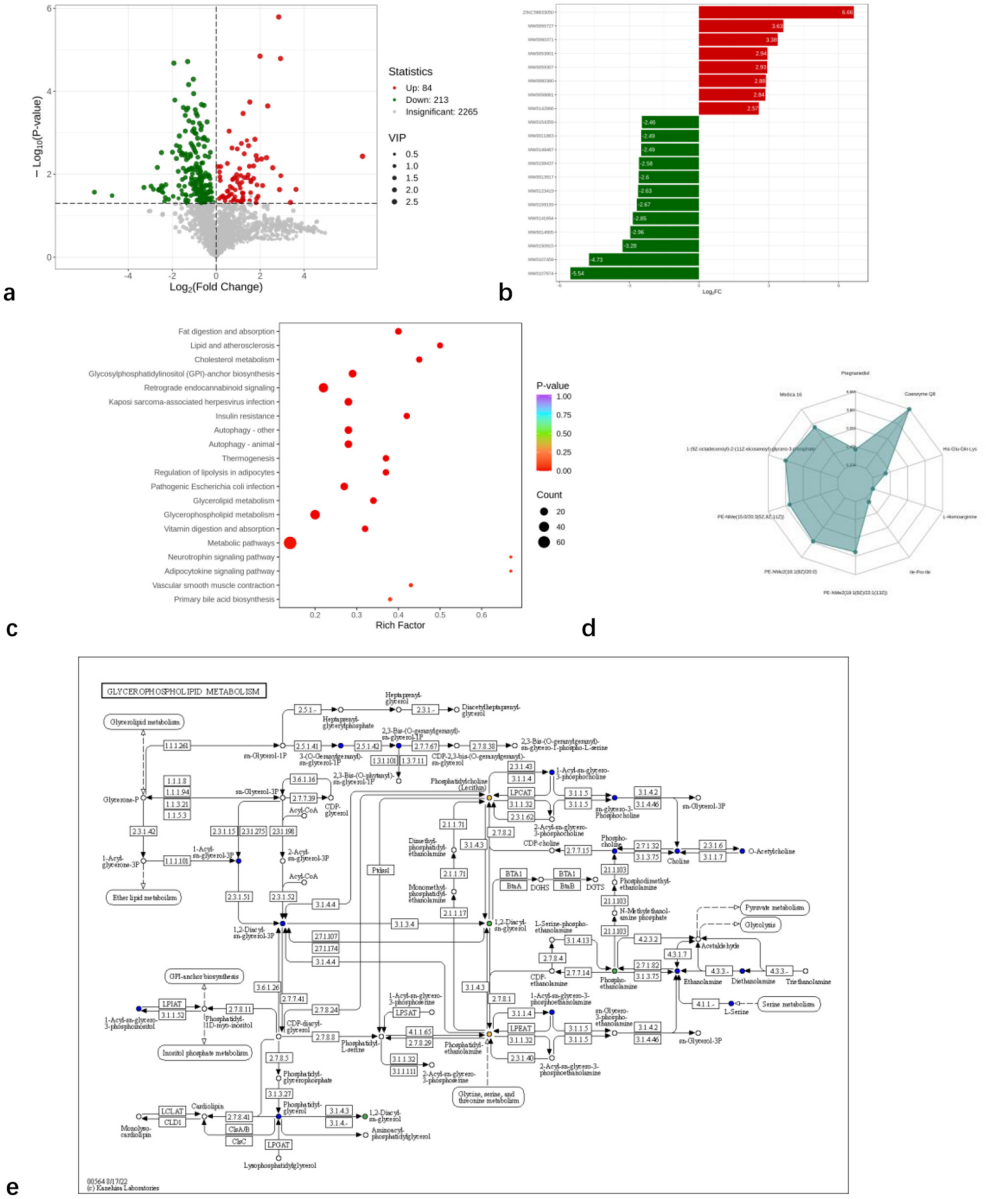
Analysis of serum metabolic differentiators and differential metabolic pathways in high and low backfat thickness Hu sheep. **(a)** volcano plot of differential metabolites; **(b)** bar plot of differential fold; **(c)** Bubble plot of differential metabolic pathways; **(d)** top Fc radar chart; **(e)** glycerophospholipid metabolism graph.

Using Student's *t*-test (*P* < 0.05) and variable importance in projection (VIP) values (VIP > 1), a total of 297 differentially expressed metabolites (84 upregulated and 213 downregulated) were identified between the two groups (Figure 3a). The analysis demonstrated significant differences in metabolite levels between the groups (Figure 3b). As shown in Figure 3c, these metabolites were primarily enriched in the following metabolic pathways: fat digestion and absorption, glycerolipid metabolism, glycerophospholipid metabolism, and primary bile acid biosynthesis. Significantly altered metabolites included 1-(9Z-octadecenoyl)-2-(11Z-eicosenoyl)-*glycero*-3-phosphate, PE-NMe (15:0/20:3[5Z,8Z,11Z]), PE-NMe2 (18:1[9Z]/20:0), PE-NMe2 (18:1[9Z]/22:1[13Z]), and Coenzyme Q8 (Figure 3d). Among these, 1-(9Z-octadecenoyl)-2-(11Z-eicosenoyl)-*glycero*-3-phosphate, PE-NMe (15:0/20:3[5Z,8Z,11Z]), PE-NMe2 (18:1[9Z]/20:0), and PE-NMe2 (18:1[9Z]/22:1[13Z]) are involved in glycerophospholipid metabolism (Figure 3e).

### 3.3 Prediction of diversity, composition, and function of intestinal bacterial communities

**Figure 4:**
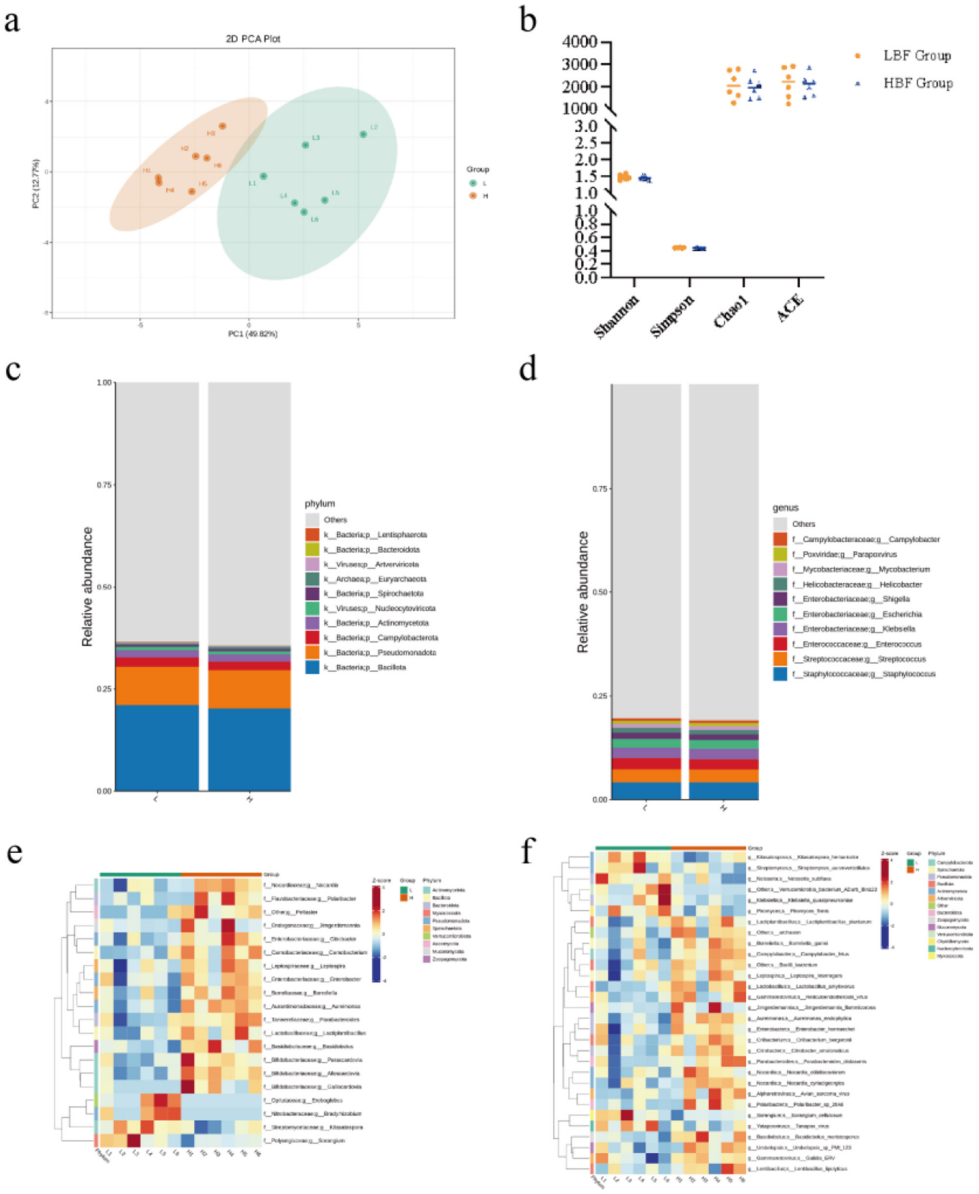
Metagenomic sequencing was used to analyze the composition and functional characteristics of the ileal microbiota of high BFT and low BFT Hu sheep. **(a)** statistical plot of microbial community diversity and total species. **(b)** PCoA principal component analysis plot. **(c)** Kingdom and phylum level community composition stacked bar chart. **(d)** family and genus level community composition stacked bar chart. **(e)** heat map of genus level and differential microorganisms. **(f)** heat map of differential strains at species level.

Gut microbiota differences among experimental groups were analyzed using metagenomic sequencing. Functional annotation of sequencing data against the KEGG database revealed functional profiles and taxonomic abundance. No significant differences in alpha diversity were observed between groups (Figure 4a). Principal coordinates analysis (PCoA) demonstrated distinct clustering of microbial community structures (Figure 4b). At the phylum level, Firmicutes, *Proteobacteria, Campylobacterota*, and *Actinobacteriota* predominated (Figure 4c). Dominant genera included *Staphylococcus, Streptococcus, Enterococcus*, and *Klebsiella* (Figure 4d). Metastats analysis identified significant inter-group differences in microbial composition and function, revealing the top 20 differentially abundant genera (Figure 4e) and top 30 differentially abundant KEGG features (Figure 4f). Notably, the HBF group exhibited significantly higher relative abundances of *Enterobacter, Parabacteroides*, and *Lactiplantibacillus* compared to the LBF group (*P* < 0.05).

**Figure 5:**
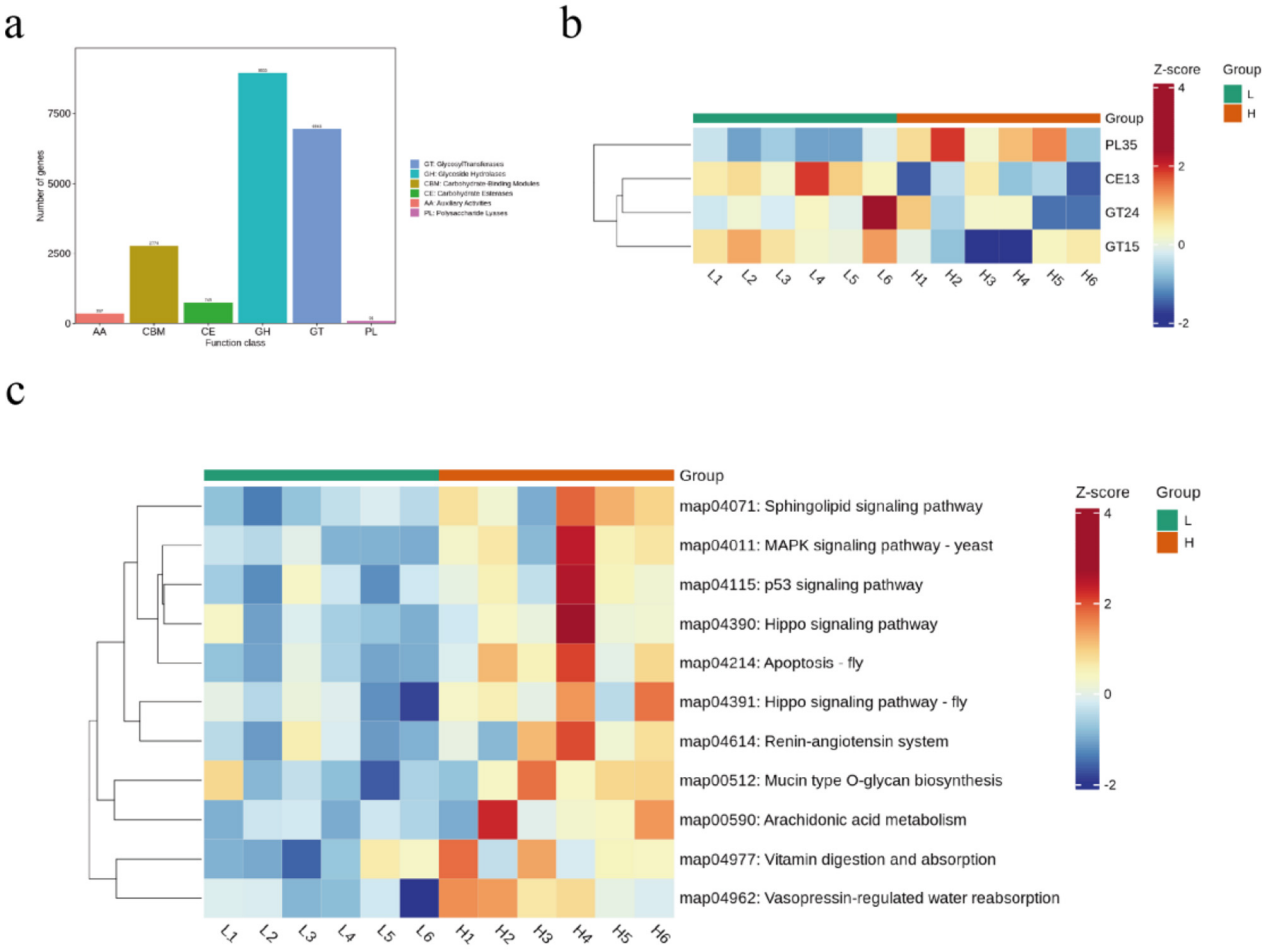
Differential analysis of microbial function in sheep from different backfat. **(a)** compositional analysis of rumen microbial enzymes. **(b)** microbial enzymes analyzed. **(c)** KEGG analyzed.

Functional profiling of the microbiome was assessed through annotation of genes encoding Carbohydrate-Active Enzymes (CAZymes). Protein sequences derived from non-redundant gene catalogs were classified into the six primary CAZyme classes. Among these, Glycoside Hydrolases (GHs) and Glycosyl Transferases (GTs) constituted the most abundant classes (Figure 5a). Notably, CAZyme families PL35, CE13, GT24, and GT15 exhibited significant differential abundance between the experimental groups (Figure 5b). KEGG pathway analysis revealed that microbial gene functions were predominantly enriched in pathways associated with lipid metabolism, including the sphingolipid signaling pathway, yeast MAPK signaling pathway, p53 signaling pathway, and arachidonic acid metabolism (Figure 5c).

### 3.4 Gut microbiota and metabolome

**Figure 6:**
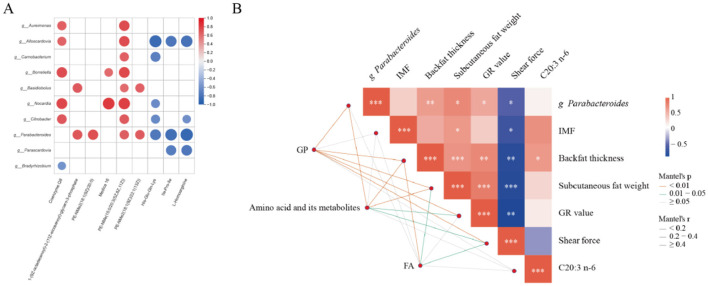
Correlation analyses. **(A)** correlation analysis between microbial metagenomes and serum metabolomes of Hu sheep with divergent backfat thickness (high vs low). **(B)** correlation analysis between *Parabacteroides* and phenotypic traits.

In order to determine the correlation between serum metabolites and gut microbiota, Spearman's correlation analysis was conducted. According to the association analysis between bacteria and lipid metabolites and fatty acids, intestinal bacteria may also affect lipid metabolism in the body to some extent. As shown in Figure 6A, *Parabacteroids* are positively correlated with 1-(9Z-octadecenoyl)-2-(11Z-eicosenoyl)-glycero-3-phosphate, PE-NMe (15:0/20:3[5Z,8Z,11Z]), PE-NMe2 (18:1[9Z]/20:0). Building upon the *Parabacteroides* species identified in Figure 6A as significantly correlated with serum metabolites, we performed Pearson correlation analysis between *Parabacteroides* and key phenotypic traits from our prior research (intramuscular fat content - IMF, backfat thickness, subcutaneous adipose tissue mass, GR value, shear force, and C20:3 n-6) (Li et al., 2025). As shown in Figure 6B, *Parabacteroides distasonis* exhibited significant positive correlations with backfat thickness, subcutaneous adipose tissue mass, and GR value, whereas a significant negative correlation was observed with meat tenderness, as measured by shear force.

## 4 Discussion

Serum biochemical indicators serve as crucial biomarkers for evaluating an organism's production performance, physiological status, and health condition (Jiao et al., 2024). HDL and LDL not only regulate normal lipid metabolism but also exhibit altered levels as key features of metabolic disorders (Durand et al., 1987). AST, primarily found in tissues such as liver and myocardium, typically elevates in serum following tissue damage or metabolic dysfunction (Mihas et al., 1981). In this study, sheep with HBF group demonstrated significantly higher HDL levels. This observation may be attributed to HDL's essential role in lipid metabolism—facilitating reverse cholesterol transport from peripheral tissues to the liver for catabolism and excretion, thereby maintaining cholesterol homeostasis (Liang et al., 2020). Concurrently, excessive adipose tissue accumulation may promote cholesterol synthesis and secretion, consequently elevating LDL levels (Aguilar et al., 2014). Furthermore, heightened lipid metabolism in high-backfat sheep could exert physiological stress on hepatic tissues, potentially explaining the observed increase in AST levels (Xie et al., 2022). Grzybowska et al. (2023) found a significant correlation between backfat thickness and blood AST concentration in fatty liver cows. This is similar to the results of this study, suggesting that high backfat thickness may increase the risk of liver health in sheep.

Phosphatidylcholine (PC) and phosphatidylethanolamine (PE) represent the primary and secondary most abundant phospholipids, respectively, with PE serving as a metabolic precursor for PC biosynthesis (Yang et al., 2018). TG and BA concentrations significantly correlate with adiposity and metabolic disorders (Gong et al., 2024; Huang et al., 2025). In the HBF cohort, levels of specific glycerophospholipids including PE (22:6[4Z,7Z,10Z,13Z,16Z,19Z]/18:1[11Z]), PE-NMe_2_ (18:2[9Z,12Z]/24:1[15Z]), and PE-NMe (15:0/20:3[5Z,8Z,11Z]) were markedly higher compared to LBF controls.This phospholipid signature suggests enhanced lipid metabolic flux in HBF sheep, potentially contributing to increased backfat deposition. Branched-chain amino acid abundance further associates with dysregulated lipid homeostasis (Lackey et al., 2013). Dietary lipid catabolism, mediated by pancreatic lipase and other hydrolases, generates absorbable metabolites for energy production and essential fatty acid provision (Omer and Chiodi, 2024). Key metabolic pathways governing these processes include glycerolipid metabolism, which regulates triacylglycerol biosynthesis/catabolism and substrate interconversion (Prentki and Madiraju, 2008); glycerophospholipid metabolism, essential for maintaining membrane phospholipid dynamics critical to cellular integrity (Hermansson et al., 2011); and primary bile acid biosynthesis, facilitating cholesterol conversion and enterohepatic circulation while demonstrating direct implications in lipid disorders when dysregulated (Cai et al., 2022). These metabolic processes are critical determinants of serum lipid profiles, with their dysregulation directly impacting circulating metabolite levels and contributing to systemic.

Gut microbiota composition and activity critically modulate obesity development (Lin et al., 2022). Notably, *Carnobacterium spp*. and *Parabacteroides distasonis* demonstrate therapeutic potential against metabolic dysregulation, attenuating weight gain, hyperglycemia, hyperlipidemia, and hepatic steatosis (Qiu et al., 2024; Li et al., 2022). Similarly, probiotic supplementation with *Bifidobacterium* spp. ameliorates obesity through metabolic reprogramming (Kim et al., 2022), while *Lactiplantibacillus* plantarum HNU082 enhances lipid catabolism and stabilizes dysbiosis during hyperlipidemia (Shao et al., 2017). Metagenomic analysis identified *Carnobacterium, Parabacteroides, Lactiplantibacillus*, and *Bifidobacterium* as the most differentially abundant genera, with *P. distasonis* exhibiting maximal species-level differentiation. These taxa potentially govern ovine lipid metabolism and backfat deposition.

PL35, a polysaccharide lyase that facilitates oligosaccharide cleavage (Lu et al., 2024), which was significantly elevated in the HBF group, suggesting enhanced oligosaccharide utilization by gut microbiota in this group. Glycosyltransferases (GTs) catalyze the synthesis of glycosidic linkages by transferring sugar residues from donor to acceptor molecules (Lairson et al., 2008). Carbohydrate Esterases (CEs) involve enzymes that catalyze de-O or de-N-acylation to remove ester decorations from carbohydrates (Cantarel et al., 2009). Carbohydrate Esterase family 13 (CE13), the smallest among CE families (Nakamura et al., 2017), along with GT24 and GT15, were significantly elevated in the LBF group. We hypothesize that CE13-mediated deacylation might affect Short-Chain Fatty Acid production, while GT24/GT15 could participate in bile acid glycosylation, modifying their fat emulsification capacity and ultimately impacting host lipid metabolism. For KEGG pathway analysis, microbial gene functions were primarily enriched in lipidogenesis-related pathways including Sphingolipid signaling pathway, MAPK signaling pathway – yeast, p53 signaling pathway, and Arachidonic acid metabolism. Collectively, these findings indicate that alterations in sheep gut microbiota composition lead to functional shifts, with lipid deposition playing a critical role in this process. However, the specific mechanistic pathways involved require further investigation.

*Parabacteroides* species generate acetic acid and succinate as primary saccharolytic metabolite (Sakamoto and Benno, 2006). Notably, *P. distasonis* supplementation ameliorates weight gain, hyperglycemia, and hepatic steatosis in metabolic disorder models (Wang et al., 2019). While the succinate/SUCNR1 signaling axis is implicated in obesity pathogenesis (Keiran et al., 2019), its association with serum metabolite profiles remains unexplored. Integrated KEGG analysis of serum metabolomics identified lipid metabolism-associated phospholipids: 1-(9Z-octadecenoyl)-2-(11Z-eicosenoyl)-glycero-3-phosphate, PE-NMe (15:0/20:3[5Z,8Z,11Z]), PE-NMe_2_ [18:1(9Z]/20:0), and PE-NMe_2_ (18:1[9Z]/22:1[13Z]). Critically, robust covariance was observed between these phospholipids and *Parabacteroides* abundance. These findings suggest *Parabacteroides* modulates host lipid homeostasis through serum glycerol-phospholipid remodeling pathways, though precise mechanistic underpinnings warrant further investigation.

## 5 Conclusion

This study preliminarily revealed the dynamic changes in gut microbiota structure and serum metabolites associated with differential backfat thickness in sheep. *Parabacteroides spp*. was identified as discriminant microorganisms distinguishing different backfat thicknesses. Key metabolites involved in glycerophospholipid metabolism included 1-(9Z-octadecenoyl)-2-(11Z-eicosenoyl)-glycero-3-phosphate, PE-NMe (15:0/20:3[5Z,8Z,11Z]), PE-NMe2 (18:1[9Z]/20:0), PE-NMe2 (18:1[9Z]/22:1[13Z]). The findings provide novel insights into the association between serum metabolites and gut microbiota in sheep with varying backfat thickness, while uncovering potential mechanisms through which microbiota may influence host lipid metabolism via metabolites. This research offers valuable references for understanding lipid metabolism regulation in ruminants. It should be noted that functional validation of *Parabacteroides distasonis* on ovine lipid metabolism has not been performed in the current work. Subsequent studies intend to conduct targeted functional verification experiments for this specific strain.

## Data Availability

The original contributions presented in the study are publicly available. This data can be found here: PRJCA044305, and publicly available datasets were analyzed in this study. This data can be found here: PRJCA044366.
